# Association between Vitamin A and E Forms and Prostate Cancer Risk in the Singapore Prostate Cancer Study

**DOI:** 10.3390/nu15122677

**Published:** 2023-06-08

**Authors:** Wei Qi Loh, Xin Yin, Rie Kishida, Sin Eng Chia, Choon Nam Ong, Wei Jie Seow

**Affiliations:** 1 Saw Swee Hock School of Public Health, National University of Singapore and National University Health System, Singapore 117549, Singapore; 2 Department of Medicine, Yong Loo Lin School of Medicine, National University of Singapore and National University Health System, Singapore 117597, Singapore

**Keywords:** vitamin forms, carotenoids, tocopherol, case-control study, mixture analysis

## Abstract

Purpose: This study aimed to assess associations between forms of vitamin A and E (both individually and collectively) and the risk of prostate cancer, as well as identify potential effect modifiers. Methods: Utilizing data from the Singapore Prostate Cancer Study, a hospital-based case-control study, we measured the serum concentrations of 15 different forms of vitamins A and E in 156 prostate cancer patients and 118 control subjects, using a high-performance liquid chromatography technique. These forms included retinol, lutein, zeaxanthin, α-cryptoxanthin, β-cryptoxanthin, α-carotene, β-carotene, lycopene, ubiquinone, δ-tocopherol, γ-tocopherol, α-tocopherol, δ-tocotrienol, γ-tocotrienol, and α-tocotrienol. The odds ratio and 95% confidence interval for associations between vitamin A and E and prostate cancer risk were estimated using logistic regression models after adjustment for potential confounders. The analyses were further stratified by smoking and alcohol consumption status. The mixture effect of micronutrient groups was evaluated using weighted quantile sum regression. Results: Higher concentrations of retinol, lutein, α-carotene, β-carotene, ubiquinone, α-tocopherol, δ-tocotrienol, γ-tocotrienol, and α-tocotrienol were significantly and positively associated with overall prostate cancer risk. Among ever-smokers, associations were stronger for lutein, β-cryptoxanthin and β-carotene compared with never-smokers. Among regular alcohol drinkers, associations were stronger for lutein, β-cryptoxanthin, ubiquinone, γ-tocotrienol and α-tocotrienol compared with non-regular alcohol drinkers. Retinol and α-tocotrienol contributed most to the group indices ‘vitamin A and provitamin A carotenoids’ and ‘vitamin E’, respectively. Conclusions: Several serum vitamin A and E forms were associated with prostate cancer risk, with significant effect modification by smoking and alcohol consumption status. Our findings shed light on prostate cancer etiology.

## 1. Introduction

Prostate cancer is characterised by uncontrolled cell growth within the prostate gland. In more than half of the countries in the world, prostate cancer is the most frequently diagnosed cancer among males [1]. In the year of 2020, there were an estimated 1,414,259 newly diagnosed cases [1]. Despite its high incidence, the etiology of this disease remains poorly understood compared with other common cancers [2]. The risk factors established to date are age, ethnicity, family history, and genetic factors [3]. Past studies have suggested that changes in diet and lifestyle may play a role in disease onset and account for temporal trends in incidence rates of prostate cancer [4]. They could also explain the differences observed in incidence rates of prostate cancer between migrant and native populations [5,6].

An essential component of our diet is micronutrients, which comprises vitamins and minerals. Micronutrients are required by the body in trace amounts and function as important co-enzymes, co-factors, or antioxidants [7]. For instance, vitamin A, also known as retinol, has been shown to modulate cell differentiation and proliferation [8]. Vitamin E—which refers to a group of four tocopherols and four tocotrienols—is a constituent of cell membranes that protects cells from oxidative damage [9]. Carotenoids, some of which may act as precursors to vitamin A (termed as ‘provitamin A’), contribute to human health through a broad spectrum of mechanisms [10]. As vitamin A and E regulate important biological processes, their involvement in prostate cancer etiology has been of great interest [11].

Recently, several epidemiological studies have investigated the association between vitamin A and E and the risk of prostate cancer by measuring the concentrations of these vitamins in plasma or serum samples: two meta-analyses and a pooled analysis of such studies found prostate cancer risk to be positively associated with circulating concentrations of retinol but inversely associated with circulating concentrations of α-tocopherol and lycopene, a non-provitamin A carotenoid [12,13,14]. Nevertheless, it remains unclear whether lifestyle factors influence the associations observed in these analyses, although studies have suggested that vitamin E supplementation may have differential effects on the prostate cancer risk of smokers and non-smokers [15]. Furthermore, most studies on prostate cancer have focused on vitamin A and E individually, and their combined effects as biologically meaningful groups may have been overlooked. There have been attempts to assess the associations between total circulating concentrations of multiple carotenoids or tocopherols with prostate cancer risk, but no study to date has collectively evaluated the effects of vitamin A and E grouped according to vitamin activity [16,17].

In addressing these research gaps, the primary objective of this study was to assess the associations between forms of vitamin A and E with prostate cancer risk in the Singapore Prostate Cancer Study (SPCS). The secondary objectives were to assess whether lifestyle factors, such as smoking and alcohol consumption, modified the observed associations and to assess the collective effect of micronutrient groups.

## 2. Materials and Methods

### 2.1. Study Population

The SPCS was a retrospective hospital-based case-control study consisting of 508 participants (240 cases and 268 controls) recruited in 2007–2009 from Singapore General Hospital. The cases were male Singaporean resident patients aged 50 to 85 years with diagnoses of incident prostate cancer confirmed by pathologists from biopsy or operative specimens. All cases were interviewed and had their blood sample collected within one month of prostate cancer diagnosis. The patients had not undergone treatment yet at the time of recruitment. The controls were patients enrolled in the same hospital under other departments with no prior history of malignant disease at the time of recruitment. The controls were frequency-matched to cases based on 5-year age groups and ethnicity. Blood samples were collected from the controls at the time of their enrollment in the study. More details can be found in the previous publication [18]. Additionally, clinical information such as the stage and grade of cancer, as well as the prostate-specific antigen (PSA) level were also collected.

In this study, we included participants for which complete data on potential confounders and sera samples for analysis were available. This resulted in a final study population of 156 cases and 118 controls.

### 2.2. Ethical Considerations

The SPCS was approved by the Institutional Review Boards of the National University of Singapore and Singapore General Hospital. The study was carried out following the Declaration of Helsinki. All participants gave informed consent to participate in the study.

### 2.3. Serum Measurements

Blood samples were collected from all the participants. The sera obtained were stored at −80 °C until analysis. Using a high-performance liquid chromatographic method, we measured the concentrations of 15 vitamin A and E: all-trans-retinol, lutein, zeaxanthin, α-cryptoxanthin, β-cryptoxanthin, α-carotene, β-carotene, lycopene, ubiquinone, δ-tocopherol, γ-tocopherol, α-tocopherol, δ-tocotrienol, γ-tocotrienol, and α-tocotrienol. The method used a mobile phase comprising acetonitrile, methanol, ethanol, and tert-butanol to separate and quantify vitamin A and E in 30µL aliquots of sera samples, which were passed through two C18 columns coupled with photodiode array, fluorescence, and electrochemical detection [19].

### 2.4. Questionnaire Measurement

Two trained research staff interviewed the participants using a standardized questionnaire that collected data on demographics (age, ethnicity, education, marital status etc.), body measurements (weight, height, etc.), family history, physical activity, sun exposure factors, as well as smoking and alcohol drinking habits.

This questionnaire included a semi-quantitative dietary section that recorded the frequency and amount at which 78 food items were consumed by participants in the past year. We obtained energy and nutrient values of these commonly eaten local food items from the local Health Promotion Board’s food composition table to estimate daily energy intake for participants in the study [20].

The participants were also interviewed regarding their eye and skin colour, as defined by skin colour on the inner upper arm. Sun exposure characteristics, skin pigment and eye pigment characteristics had previously been associated with prostate cancer risk in this population [18].

### 2.5. Statistical Analysis

The enrolment characteristics of case and controls were compared as categorical variables with the Chi-squared test or Fisher’s exact test. Serum vitamin A and E concentrations between cases and controls were compared using the Wilcoxon signed-rank test as they were not normally distributed. Using unconditional logistic regression models, we estimated the odds ratios (ORs) of prostate cancer risk and the respective 95% confidence intervals (CIs) according to the tertiles or median values of serum micronutrient concentrations in controls as cut-offs. We used median values as cut-offs when categorisation by tertile values resulted in any category containing <10% of the cases. This was to prevent extremely wide confidence intervals. There were two reasons for there being such a small number of cases or controls in specific tertile groups: (1) The median values were based on control distributions. Therefore, imbalanced distributions were particularly observed among cases. (2) For some vitamin A and E compounds, there were many observations at a certain value. Therefore, the tertiles did not contain equally distributed categories of controls in three parts. For example, it was not possible to split a-carotene into tertiles as many patients had the same concentration values. In all our analyses, the lowest micronutrient category was taken as the referent.

We adjusted for characteristics that differed significantly between cases and controls in the model: age (continuous), years of education (never, 1–6 years, 7–10 years, >10 years), family history of any cancers (yes or no), body mass index (BMI) (continuous) [21], sun exposure frequency (never, seldom, occasionally, frequent), skin pigment (very white/white, light tan, tan/dark brown/black), and eye pigment characteristics (black/dark brown, light brown) [18]. Tests for linear trends (*P*-trend) across tertiles of serum vitamin A and E concentrations were based on an ordinal variable according to rank, from lowest to highest. In our exploratory analyses, we carried out a sensitivity analysis that further adjusted for daily energy intake.

To assess effect modification, we stratified the analyses according to smoking and alcohol consumption status. Smoking status was evaluated based on participant reports of having ever smoked a cigarette in their lifetime, while regular alcohol consumption status was evaluated based on participant reports of having ever drunk alcohol more than once a month on average. We also included a cross-product term of each serum vitamin A and E concentration (on a continuous scale) and the modifier variable (ever-smoker vs. never-smoker, regular alcohol drinker vs. non-regular alcohol drinker) in the regression model and evaluated the significance of this cross-product term (*P*-interaction) using Wald’s test.

The correlations between serum concentrations of individual vitamin A and E were evaluated by Pearson’s correlation coefficient, as denoted by r. To carry out mixture analysis and estimate the collective effect of multiple vitamin A and E on prostate cancer risk, we performed weighted quantile sum (WQS) regression using the ‘gWQS’ package in R [22]. Here, we estimated the association of two a priori-defined micronutrient groups (‘vitamin A and provitamin A carotenoids’ which included retinol, α-cryptoxanthin, β-cryptoxanthin, α-carotene, and β-carotene; and ‘vitamin E’ which included δ-tocopherol, γ-tocopherol, α-tocopherol, δ-tocotrienol, γ-tocotrienol, and α-tocotrienol) in relation to prostate cancer risk. The weight of a micronutrient, derived from bootstrapping, indicated how much an individual micronutrient contributed to the index. This allowed us to rank the vitamin A and E in order of importance to the overall risk estimate. We chose to score the indices by deciles and carry out 100 bootstrap samples in our models. Due to the small sample size, we did not split the data into a training and validation set. We also carried out further exploratory analysis that categorised prostate cancer by tumor stage and grade.

Analyses were performed using Rstudio (version 1.3.1073) implementing R software (version 4.0.2). All statistical tests were two-sided, with a *p* value of <0.05 considered as significant. The statistical significance of associations between individual vitamin A and E and overall prostate cancer risk were further adjusted for multiple testing using the Benjamini–Hochberg method.

## 3. Results

### 3.1. Baseline Characteristics of Cases and Controls

In this study, prostate cancer cases were more likely to be older and have a higher educational level, first degree relatives with history of any cancer, lower BMI, black/dark brown eyes, tanner skin, and a higher frequency of sunburn exposure (Table 1) than controls. No significant differences were detected between cases and controls for ethnicity, marital status, smoking and alcohol consumption status. Other clinical characteristics, including stage and grade of cancer and concentration of prostate-specific antigen (PSA), are presented in Supplementary Table S1.

### 3.2. Serum Vitamin A and E in Cases and Controls

We compared the serum concentrations of 15 forms of vitamin A and E between the cases and controls (Table 2). The concentrations of retinol, lutein, β-cryptoxanthin, α-carotene, β-carotene, ubiquinone, α-tocopherol, δ-tocotrienol, γ-tocotrienol, and α-tocotrienol were significantly higher in the cases compared with the controls. On the other hand, the concentration of γ-tocopherol was lower among cases than in controls, but the difference was not statistically significant.

### 3.3. Associations of Individual Serum Vitamin A and E with Prostate Cancer Risk

Table 3 presents the associations of micronutrient concentrations with prostate cancer risk according to tertiles or median values of controls as cut-offs. The highest category of serum concentrations for several forms of vitamin A and E were significantly and positively associated with prostate cancer risk, including retinol (OR = 6.08), lutein (OR = 2.07), α-carotene (OR = 3.27), β-carotene (OR = 3.04), ubiquinone (OR = 2.44), α-tocopherol (OR = 2.40), δ-tocotrienol (OR = 3.70), γ-tocotrienol (OR = 3.02), and α-tocotrienol (OR = 2.76), compared with the lowest category. These associations remained significant even after adjusting for multiple comparisons (Benjamini–Hochberg method). There was a significant increasing trend in prostate cancer risk according to tertiles of ubiquinone, α-tocopherol, δ-tocotrienol, γ-tocotrienol, and α-tocotrienol concentrations. Although serum lycopene (OR = 0.92) and γ-tocopherol (OR = 0.60) in the highest tertile were inversely associated with prostate cancer risk, these were not statistically significant. Similar results were observed when the models were further adjusted for energy intake (Supplementary Table S2). In the exploratory analyses, the associations were similar across tumor grade (Supplementary Table S3) and stage (Supplementary Table S4).

### 3.4. Smoking and Alcohol Consumption Status as Effect Modifiers

As shown in Table 4, smoking status was a significant effect modifier for the associations of lutein, β-cryptoxanthin and β-carotene with prostate cancer risk. The associations were stronger among ever-smokers compared with never-smokers for the highest concentrations of lutein (ever-smokers OR = 6.43 vs. never-smokers OR = 2.75), β-cryptoxanthin (ever-smokers OR = 7.50 vs. never-smokers OR = 1.08), and β-carotene (ever-smokers OR = 5.61 vs. never-smokers OR = 1.88).

In addition, alcohol consumption status was observed to significantly modify the associations of lutein, β-cryptoxanthin, ubiquinone, γ-tocotrienol and α-tocotrienol concentrations with prostate cancer risk (Table 4). The associations were stronger among those who had drunk alcohol regularly before compared with those who had not for the highest concentrations of lutein (regular alcohol drinkers OR = 2.71 vs. non-regular alcohol drinkers OR = 1.81), β-cryptoxanthin (regular alcohol drinkers OR = 2.96 vs. non-regular alcohol drinkers OR = 1.28), ubiquinone (regular alcohol drinkers OR = 3.35 vs. non-regular alcohol drinkers OR = 1.99), γ-tocotrienol (regular alcohol drinkers OR = 3.39 vs. non-regular alcohol drinkers OR = 2.71), and α-tocotrienol (regular alcohol drinkers OR = 3.06 vs. non-regular alcohol drinkers OR = 1.46).

### 3.5. Mixture Analysis with Multiple Vitamin A and E

Prior to performing WQS regression, we tested for correlations between vitamin A and E (Supplementary Table S5). The concentrations of the four provitamin A carotenoids: α-cryptoxanthin, β-cryptoxanthin, α-carotene, β-carotene were significantly and positively correlated with one other, with modest to strong correlation coefficients (r ranging from 0.25 to 0.79). Apart from δ-tocotrienol with γ-tocopherol and α-tocopherol, the concentrations of the six vitamin E isoforms were otherwise significantly and positively correlated with one another, with modest to strong correlation coefficients (r ranging from 0.11 to 0.73).

The results of the WQS regression model, which allows us to account for collinearity, are presented in Table 5. Two a priori-defined empirically-weighted indices, ‘vitamin A and provitamin A carotenoids’ and ‘vitamin E’, were positively associated with prostate cancer risk, with an increase of 65% and 47% in risk estimates observed for every decile increase in the index concentrations, respectively.

**Figure 1:**
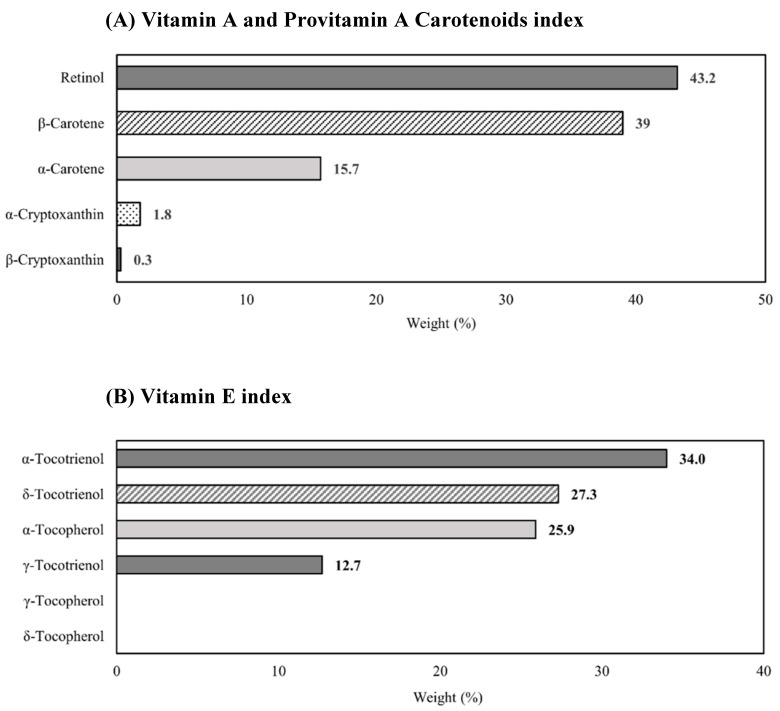
Estimated weights for vitamin A and E components in weighted quantile sum indices.

As shown in Figure 1, retinol had the greatest weight (43.2%), followed by β-carotene (39.0%) and α-carotene (15.7%) for the WQS regression index ‘vitamin A and provitamin A carotenoids’. Meanwhile, α-tocotrienol, δ-tocotrienol and α-tocopherol were the top three contributors, with weights of 34.0%, 27.3% and 25.9%, respectively, for the ‘vitamin E’ index (Figure 1). β-cryptoxanthin, γ-tocopherol and δ-tocopherol contributed minimally to their respective indices, with weights of less than 1.0%.

## 4. Discussion

Our study investigated the associations between 15 different forms of vitamin A and E and prostate cancer risk in Singapore. Among these forms, higher concentrations of serum retinol, lutein, α-carotene, β-carotene, ubiquinone, α-tocopherol, δ-tocotrienol, γ-tocotrienol, and α-tocotrienol were significantly and positively associated with prostate cancer risk. Specifically, retinol and α-tocotrienol contributed most to the group indices ‘vitamin A and provitamin A carotenoids’ and ‘vitamin E’, respectively.

For retinol, our findings are largely consistent with the existing literature. A collaborative pooled analysis including the data of 29,780 participants from 15 prospective studies previously reported a significant positive association between pre-diagnostic circulating concentrations of retinol and prostate cancer risk [12]. Positive associations were also replicated in two updated nested case-control studies: one from the Alpha-Tocopherol-Beta-Carotene trial which investigated the use of α-tocopherol and β-carotene supplements among smokers, and one from the placebo arm of the Prostate Cancer Prevention Trial (PCPT) which examined the effect of prostate-specific antigen screening for prostate cancer in healthy adult men [23,24,25]. Recently, a study showed that genetic variants (rs1330286 and rs4646653) involved in retinol metabolism pathways were significantly associated with prostate cancer risk [26]. This further serves as evidence that retinol may be involved in prostate cancer development, and further studies will be useful to characterise the nature of this relationship.

Studies investigating the relationship between vitamin E concentrations and prostate cancer risk have generally focused on the isoforms α-tocopherol and γ-tocopherol. Two meta-analyses have suggested an inverse association between circulating concentrations of α-tocopherol with prostate cancer risk, but null associations between circulating concentrations of γ-tocopherol with prostate cancer risk [12,14]. In our study, we similarly observed a null association for γ-tocopherol but detected a modest positive association for α-tocopherol with prostate cancer risk. Additionally, we found significant positive associations of δ-tocotrienol, γ-tocotrienol and α-tocotrienol concentrations with prostate cancer risk. To our knowledge, studies have yet to investigate the relationship between serum concentrations of tocotrienols and prostate cancer risk. Previous studies measuring tocotrienol status have only done so through self-reported dietary intake but found null associations with prostate cancer risk [27,28]. Tocotrienols are naturally occurring compounds in vegetable oils such as palm oil and rice brain oil, as well as wheat germ and barley. The associations we found between serum tocotrienols and prostate cancer risk in this study may provide potential leads for future research on tocotrienols.

Among the carotenoids, circulating concentrations of lycopene have previously been reported to be inversely associated with prostate cancer risk in a meta-analysis [13]. Although we obtained an OR of <1.0 for prostate cancer risk among those with higher serum concentrations of lycopene, this association did not reach statistical significance in our study. We acknowledge that our study may have been underpowered to detect the magnitude of difference due to the small sample size. The positive association we observed between serum α-carotene and prostate cancer risk was consistent with the findings of a previous nested case-control analysis from PCPT, and the positive associations for serum lutein and β-carotene with prostate cancer risk in our study were concordant with a recent study conducted among low-income African and European American males [25,29]. Interestingly, the latter study showed that only the trans-isomers, and not the cis-isomers, of lutein and β-carotene were significantly associated with prostate cancer risk [29]. However, the effect of geometric isomerism could not be clarified in our study as our measurement method did not distinguish between isomers. For most of the other carotenoids (zeaxanthin, α-cryptoxanthin, β-cryptoxanthin), nested case-control studies from large-scale randomized trials and cohort studies have reported null associations between serum concentrations and prostate cancer risk in healthy adult males, which was consistent with our results [25,29,30,31,32].

Ubiquinone, also known as coenzyme Q10, is reported to exert anti-oxidative effects distinct from those of vitamin E [33]. Although its potential to mitigate the risk of chronic diseases (e.g., cardiovascular disease and cancer) has been of great research interest, the body of evidence for the role of ubiquinone in prostate cancer is limited [34]. To the best of our knowledge, there has been only one nested case-control study from the Multi-ethnic Cohort in the United States, which determined that there was no significant association of plasma concentrations of ubiquinone with prostate cancer risk [35]. In contrast, we observed a significant and positive association between serum ubiquinone concentrations and prostate cancer risk, with ORs that were more pronounced for high-grade and advanced prostate cancer. More observational data would be needed before drawing further conclusions regarding this relationship.

A possible reason for the general differences between our findings and the existing literature is study design. Most studies that use serum or plasma samples have been prospective in nature, whereas the SPCS is a case-control study. We acknowledge that it is difficult to distinguish the temporal sequence of events (i.e., micronutrient concentrations and disease state) in this study, and the associations observed could be due to reverse causation. In addition, the differences in the ethnicity distribution in our study and the small sample size may partially explain this difference. It has been postulated that α-tocopherol may interact with the action of sex steroid hormones, which have been implicated in prostate cancer development and treatment [36].

In this study, we found evidence for significant effect modification by smoking status on the positive associations of lutein, β-cryptoxanthin and β-carotene with prostate cancer risk; as well as by alcohol consumption status on the positive associations of lutein, β-cryptoxanthin, ubiquinone, γ-tocotrienol and α-tocotrienol with prostate cancer risk. There has been limited research on the influence of lifestyle factors in such settings; the few studies examining these influences have found null associations [12,23]. The magnitude of association was greater in ever-smokers than never-smokers, and in regular alcohol drinkers than non-regular alcohol drinkers. As smoking and alcohol consumption are not established risk factors of prostate cancer, and the cases and controls did not differ significantly in these characteristics, the results we observed are likely to be of effect modification than confounding. Cigarette smoke and alcohol consumption has been reported to accelerate the degradation of micronutrients such as carotenoids; the harmful breakdown products generated may represent one possible way through which interactions contribute to increased prostate cancer risk [37,38,39]. For instance, the breakdown of β-carotene is known to generate epoxides, aldehydes and apo-carotenals, which have been shown to interfere with retinoid signaling that is essential for cell differentiation, proliferation, and apoptosis [40,41,42,43].

One key finding of our study was that specific forms of vitamin A and provitamin A carotenoids, as well as vitamin E, were more strongly associated with prostate cancer risk than others. Retinol, followed by β-carotene, contributed most to the positive association for the ‘vitamin A and provitamin A carotenoids’ index. Pertinently, retinol is the most biologically active form of vitamin A, and β-carotene is the most efficient provitamin A compared with the others due to its unique structure and cleavage efficacy [41,42,43]. The relationship observed with prostate cancer risk may possibly be related to the vitamin A activity of these two micronutrients. For vitamin E, α-tocotrienol and δ-tocotrienol contributed more weight to the ‘vitamin E’ index than α-tocopherol, which has been the most extensively studied among the isoforms. This suggests there could be value in investigating the biological activities of tocotrienols. Fewer clinical studies have been conducted on tocotrienols than on tocopherols [44]. Although less abundant than α-tocopherol in the human body, tocotrienols may exert unique biological effects [45]. We note that as our study did not include measurements of β-tocopherol and β-tocotrienol, the association we observed between the ‘vitamin E’ index and risk may not be wholly representative of the effect of the entire group of vitamin E compounds. We found prostate cancer risk was positively associated with α-tocopherol, but not with δ-tocopherol or γ-tocopherol in this study. One possible explanation is that δ-tocopherol and γ-tocopherol have different biological activities [46]. Furthermore, it has been established that the concentrations of α-tocopherol and γ-tocopherol are inversely related [46,47]. On the other hand, δ-tocopherol is present at low amounts in the human body, and its biological activities remain unclear compared with γ-tocopherol [48]. As such, the null association detected in our study is unsurprising. Previous studies have also indicated that prostate cancer risk is often associated (albeit inversely) with α-tocopherol levels but not the other tocopherols.

To our knowledge, this is the first study to evaluate the collective impact of retinol and provitamin A carotenoids, as well as vitamin E isoforms, as biologically meaningful groups on prostate cancer risk using WQS regression. This analytical technique allows us to assess the effect of chemical mixtures as empirically weighted indices on a health outcome, where traditional regression methods may face challenges due to the high correlations within or between classes of metabolites/contaminants [49]. WQS regression has mostly been used to evaluate the mixture effect of pollutants and/or contaminants in environmental health studies but was also recently used to evaluate the overall effect of dietary vitamin B forms on arsenic metabolism and preclinical markers of cardiovascular disease [50]. We are anticipating more studies in the field of nutritional epidemiology to adopt this analytical technique in the future. Nutritional biomarkers, particularly those derived from shared dietary sources and metabolic pathways, tend to be highly correlated, and WQS regression enables us to both evaluate their overall effect as a group and rank individual effects despite collinearity.

One strength of this study is the use of serum vitamin A and E concentrations as a measure of exposure status. Serum vitamin A and E allow for an objective reading of micronutrient status without the recall biases and measurement errors that may occur with food frequency questionnaires [51]. Although we make use of measurements made at a single timepoint, a previous study suggests that intraindividual variability for serum measures of micronutrients over time is low and that a single measurement can provide an estimate within 20% of the true value for most micronutrients [52]. We note that as correlations between dietary intake and serum/plasma concentrations of micronutrients may be weak, our results could be reflecting the relationship between metabolic status, rather than that of diet, with prostate cancer risk [31,53]. Another strength of our study is the use of a multi-ethnic Asian study population from Singapore. Our findings, therefore, complement previous studies, which have been limited to Western populations from America, Europe, and Canada.

We acknowledge that there are several limitations with our study. Firstly, the hospital-based case-control study design is unable to prove a temporal relation between serum vitamin A and E and prostate cancer risk and may be subject to selection bias. Hospitalized cancer patients may tend to be more severe cases, and the control group may have some diseases related to serum vitamin A and E, which may alter our results. Secondly, the small sample size in our study provides limited statistical power and precision of the analysis, and may result in associations attributable to chance. Although the findings in our main study population remain robust even after correcting for multiple testing, we acknowledge that associations in the stratified analyses are exploratory in nature due to the reduced sample size and the case-control study design. Thirdly, the WQS regression method constrains the exposure-outcome relation to a unidirectional effect. As both group indices ‘vitamin A and provitamin A carotenoids’ and ‘vitamin E’ were positively associated with risk, any inverse associations of vitamin A and E components with risk would not have been detected. This is unlikely to have affected our results much as the associations of vitamin A and E components with prostate cancer risk, when evaluated individually, were either null or significantly positive in this study. Fourthly, due to missing information on the total number of cigarettes and alcohol consumed, residual confounding may be present.

In light of the limitations outlined above, future research should employ a large-scale prospective cohort design to better establish a temporal link between serum vitamin A and E levels and the risk of prostate cancer. A thorough collection of data on lifestyle factors such as smoking and alcohol consumption can help reduce residual confounding. It would also be beneficial to conduct mechanistic studies to shed light on the precise biological pathways that connect these serum vitamin levels to prostate cancer risk. Incorporating multi-center studies would enhance the generalizability of findings. Additionally, future research should include larger, more extensive studies spanning various populations to determine if these findings can be replicated.

## 5. Conclusions

In conclusion, we detected significant positive associations of serum retinol, lutein, α-carotene, β-carotene, ubiquinone, α-tocopherol, δ-tocotrienol, γ-tocotrienol, and α-tocotrienol concentrations with prostate cancer risk. In general, the magnitude of the association was greater in ever-smokers and those who regularly consumed alcohol, suggesting that lifestyle factors may act as effect modifiers. Specific forms of vitamin A or vitamin E (retinol and α-tocotrienol, respectively) were most strongly associated with prostate cancer risk. Our findings support existing evidence regarding the involvement of vitamin A and E in prostate cancer risk. Future mechanistic studies will be useful to clarify the biological pathways by which these serum vitamin A and E are related to prostate cancer risk.

## Figures and Tables

**Table 1:** Characteristics of cases and controls from the Singapore Prostate Cancer Study.

Characteristics	Cases (*n* = 156)	Controls (*n* = 118)	*p* Value ^a^
Age			**1.1 × 10^−5^**
50–59 years old	33 (21.2)	57 (48.3)	
60–69 years old	80 (51.3)	37 (31.4)	
70 years and above	43 (27.6)	24 (20.3)	
Ethnicity			0.18
Chinese	140 (89.7)	99 (81.4)	
Malay	6 (3.8)	9 (6.8)	
Indian	8 (5.1)	14 (11.0)	
Others	2 (1.3)	2 (0.8)	
Education			**0.0011**
Never	3 (1.9)	5 (4.2)	
1–6 years	28 (17.9)	33 (28.0)	
7–10 years	55 (35.3)	54 (45.8)	
>10 years	70 (44.9)	26 (26.0)	
Marital Status			0.13
Currently married	147 (94.2)	104 (88.1)	
Separated/ Widowed	4 (2.6)	9 (7.6)	
Never married	5 (3.2)	5 (4.2)	
Family history of cancer in first degree relatives			**6.4 × 10^−5^**
No	84 (53.8)	92 (78.0)	
Yes	72 (46.2)	26 (22.0)	
Body mass index (kg/m^2^)			**0.0096**
Quartile 1 (<22.0)	43 (27.6)	29 (24.6)	
Quartile 2 (22.0–24.9)	63 (40.4)	29 (24.6)	
Quartile 3 (25.0–27.9)	30 (19.2)	33 (28.0)	
Quartile 4 (≥28.0)	20 (12.8)	27 (22.9)	
Ever-smoker			0.94
No	88 (56.4)	64 (55.2)	
Yes	68 (43.6)	52 (44.8)	
Regular alcohol consumption			0.21
No	81 (53.3)	50 (44.6)	
Yes	71 (46.7)	62 (55.4)	
Eye colour			**1.28 × 10^−8^**
Light brown	13 (8.3)	44 (37.3)	
Black/dark brown	143 (91.7)	74 (62.7)	
Skin colour			**0.0012**
Very white/white	15 (9.6)	31 (26.3)	
Light tan	86 (55.1)	52 (44.1)	
Tan/dark Brown/black	55 (35.3)	35 (29.7)	
Sunburn exposure			**0.0017**
Never	65 (41.7)	69 (58.5)	
Seldom	39 (25.0)	33 (28.0)	
Occasionally	24 (15.4)	9 (7.6)	
Frequently	28 (17.9)	7 (5.9)	

Values for baseline characteristics are reported as *n* (%). ^a^ Differences in characteristics were compared using the chi-squared test for all variables except for ethnicity, which was compared using the Fischer’s exact test. Bold values refer to statistically significant results with *p* < 0.05.

**Table 2:** Serum micronutrient concentrations of participants from the Singapore Prostate Cancer Study.

	Serum Concentrations (µg/dL)						*p* Value
	Total (*n* = 274)		Cases (*n* = 156)		Controls (*n* = 118)		
	Mean (SD)	Median (IQR)	Mean (SD)	Median (IQR)	Mean (SD)	Median (IQR)	
Retinol	75.3 (26.8)	73.5 (34.9)	83.1 (23.5)	82.4 (28.9)	65.0 (27.6)	60.9 (38.3)	**4.1 × 10** **^−9^**
Lutein	17.0 (9.0)	15.4 (10.6)	19.8 (9.5)	17.4 (11.1)	13.3 (6.6)	11.8 (7.6)	**4.4 × 10** **^−11^**
Zeaxanthin	4.6 (2.0)	4.2 (2.1)	4.6 (1.8)	4.3 (2.0)	4.6 (2.3)	4.0 (2.5)	0.27
α-Cryptoxanthin	88.0 (79.2)	67.4 (87.7)	96.3 (88.1)	71.8 (94.3)	77.1 (64.3)	57.3 (76.6)	0.15
β-Cryptoxanthin	19.4 (18.9)	13.3 (17.8)	22.9 (22.2)	14.2 (19.7)	14.8 (11.9)	11.0 (12.4)	**0.0012**
α-Carotene	1.0 (1.0)	0.5 (0.7)	1.2 (1.2)	0.7 (1.0)	0.7 (0.7)	0.5 (0.1)	**5.3 × 10** **^−8^**
β-Carotene	9.2 (8.9)	6.7 (8.3)	11.8 (9.2)	9.6 (10.1)	5.9 (7.2)	4.2 (4.6)	**5.3 × 10** **^−13^**
Lycopene	7.1 (5.2)	5.6 (6.0)	7.4 (5.4)	5.9 (5.8)	6.8 (5)	5.3 (5.9)	0.23
Ubiquinone	41.7 (18.5)	38.2 (23.8)	44.7 (20.2)	41.8 (23.4)	37.8 (15.3)	35.8 (22.0)	**0.0043**
δ-Tocopherol	11.9 (5.3)	11.1 (6.0)	11.9 (5.6)	11.2 (6.4)	11.9 (4.9)	11.0 (5.6)	0.76
γ-Tocopherol	61.4 (29.5)	57.8 (39.9)	58.9 (27.6)	55.4 (34.7)	64.8 (31.5)	60.6 (43.5)	0.10
α-Tocopherol	1560.4 (508.2)	1497.7 (541.5)	1669.5 (565.3)	1569.1 (540.6)	1416.1 (377.3)	1402.6 (504.5)	**5.9 × 10** **^−5^**
δ-Tocotrienol	1.3 (1.1)	1.1 (0.7)	1.5 (1.1)	1.3 (0.8)	1.2 (1.2)	0.9 (0.6)	**7.2 × 10** **^−6^**
γ-Tocotrienol	1.9 (2.2)	1.3 (1.3)	2.3 (2.8)	1.5 (1.7)	1.3 (0.8)	1.2 (1.0)	**0.00030**
α-Tocotrienol	1.9 (1.7)	1.3 (1.4)	2.3 (2.1)	1.6 (1.9)	1.4 (1)	1.1 (1)	**0.00018**

Serum micronutrient concentrations of cases and controls were compared using the Wilcoxon signed-rank test. IQR, interquartile range; SD, standard deviation. Bold values refer to statistically significant results with *p* < 0.05.

**Table 3:** Associations between individual serum vitamin A and E and prostate cancer risk.

	Cases (*n* = 156)	Controls (*n* = 118)		
	*n* (%)	*n* (%)	Crude OR (95% CI)	Adjusted OR (95% CI) ^a^
Retinol				
≤Median of 60.9 µg/dL	20 (12.8)	59 (50.0)	1.00 (Ref)	1.00 (Ref)
>Median of 60.9 µg/dL	136 (87.2)	59 (50.0)	6.80 (3.76–12.30)	6.08 (2.80–13.20)
Lutein				
≤Median of 11.8 µg/dL	34 (21.8)	59 (50.0)	1.00 (Ref)	1.00 (Ref)
>Median of 11.8 µg/dL	122 (78.2)	59 (50.0)	3.59 (2.12–6.06)	2.07 (1.07–4.02)
Zeaxanthin				
Tertile 1 (≤3.3 µg/dL)	34 (21.8)	41 (34.7)	1.00 (Ref)	1.00 (Ref)
Tertile 2 (3.3–4.9 µg/dL)	67 (42.9)	38 (32.2)	2.13 (1.16–3.89)	1.35 (0.63–2.92)
Tertile 3 (>4.9 µg/dL)	55 (35.3)	39 (33.1)	1.70 (0.92–3.14)	1.32 (0.59–2.96)
		*P* for trend ^b^	0.11	0.52
α-Cryptoxanthin				
Tertile 1 (≤44.7 µg/dL)	45 (28.8)	40 (34.2)	1.00 (Ref)	1.00 (Ref)
Tertile 2 (44.7–82.9 µg/dL)	43 (27.6)	38 (32.5)	1.01 (0.55–1.85)	1.23 (0.55–2.73)
Tertile 3 (>82.9 µg/dL)	68 (43.6)	39 (33.3)	1.55 (0.87–2.77)	1.93 (0.91–4.08)
		*P* for trend ^b^	0.13	0.084
β-Cryptoxanthin				
Tertile 1 (≤7.8 µg/dL)	29 (18.6)	40 (33.9)	1.00 (Ref)	1.00 (Ref)
Tertile 2 (7.8–16.0 µg/dL)	56 (35.9)	39 (33.1)	1.98 (1.06–3.71)	1.80 (0.80–4.05)
Tertile 3 (>16.0 µg/dL)	71 (45.5)	39 (33.1)	2.51 (1.35–4.66)	1.96 (0.89–4.32)
		*P* for trend ^b^	**0.0045**	0.11
α-Carotene				
≤Median of 0.50 µg/dL	64 (41.0)	86 (72.9)	1.00 (Ref)	1.00 (Ref)
>Median of 0.50 µg/dL	92 (59.0)	32 (27.1)	3.86 (2.31–6.47)	3.27 (1.67–6.38)
β-Carotene				
≤Median of 4.2 µg/dL	30 (19.2)	60 (50.8)	1.00 (Ref)	1.00 (Ref)
>Median of 4.2 µg/dL	126 (80.8)	58 (49.2)	4.34 (2.54–7.44)	3.04 (1.53–6.02)
Lycopene				
Tertile 1 (≤3.9 µg/dL)	47 (30.3)	40 (34.5)	1.00 (Ref)	1.00 (Ref)
Tertile 2 (3.9–7.5 µg/dL)	44 (28.4)	38 (32.8)	0.99 (0.54–1.80)	0.96 (0.44–2.13)
Tertile 3 (>7.5 µg/dL)	64 (41.3)	38 (32.8)	1.43 (0.80–2.57)	0.92 (0.43–1.97)
		*P* for trend ^b^	0.22	0.83
Ubiquinone				
Tertile 1 ≤ (29.3 µg/dL)	32 (20.5)	40 (34.5)	1.00 (Ref)	1.00 (Ref)
Tertile 2 (29.3–41.2 µg/dL)	45 (28.8)	37 (31.9)	1.52 (0.80–2.87)	1.27 (0.57–2.82)
Tertile 3 (>41.2 µg/dL)	79 (50.6)	39 (33.6)	2.53 (1.39–4.63)	2.44 (1.13–5.28)
		*P* for trend ^b^	**0.0022**	**0.020**
δ-Tocopherol				
Tertile 1 (≤9.7 µg/dL)	59 (37.8)	40 (33.9)	1.00 (Ref)	1.00 (Ref)
Tertile 2 (9.7–13.1 µg/dL)	45 (28.8)	39 (33.1)	0.78 (0.44–1.41)	0.971 (0.46–2.04)
Tertile 3 (>13.1 µg/dL)	52 (33.3)	39 (33.1)	0.90 (0.51–1.61)	1.08 (0.51–2.28)
		*P* for trend ^b^	0.72	0.84
γ-Tocopherol				
Tertile 1 (≤47.2 µg/dL)	54 (34.6)	41 (34.7)	1.00 (Ref)	1.00 (Ref)
Tertile 2 (47.2–74.9 µg/dL)	66 (42.3)	38 (32.2)	1.32 (0.75–2.33)	1.72 (0.80–3.70)
Tertile 3 (>74.9 µg/dL)	36 (23.1)	39 (33.1)	0.70 (0.38–1.29)	0.60 (0.28–1.31)
		*P* for trend ^b^	0.30	0.24
α-Tocopherol				
Tertile 1 (≤1240 µg/dL)	26 (16.7)	40 (33.9)	1.00 (Ref)	1.00 (Ref)
Tertile 2 (1240–1560 µg/dL)	51 (32.7)	39 (33.1)	2.01 (1.05–3.84)	1.71 (0.75–3.87)
Tertile 3 (>1560 µg/dL)	79 (50.6)	39 (33.1)	3.12 (1.67–5.82)	2.40 (1.08–5.35)
		*P* for trend ^b^	**0.00041**	**0.034**
δ-Tocotrienol				
Tertile 1 (≤0.7 µg/dL)	26 (16.7)	42 (35.6)	1.00 (Ref)	1.00 (Ref)
Tertile 2 (0.7–1.1 µg/dL)	39 (25.0)	43 (36.4)	1.47 (0.76–2.82)	1.33 (0.58–3.04)
Tertile 3 (>1.1 µg/dL)	91 (58.3)	33 (28.0)	4.45 (2.37–8.37)	3.70 (1.66–8.25)
		*P* for trend ^b^	**1.3 × 10^−6^**	**0.00079**
γ-Tocotrienol				
Tertile 1 (≤0.9 µg/dL)	43 (27.6)	41 (34.7)	1.00 (Ref)	1.00 (Ref)
Tertile 2 (0.9–1.5 µg/dL)	38 (24.4)	43 (36.4)	0.84 (0.48–1.55)	0.992 (0.45–2.2)
Tertile 3 (>1.5 µg/dL)	75 (48.1)	34 (28.8)	2.10 (1.17–3.79)	3.02 (1.38–6.59)
		*P* for trend ^b^	**0.0098**	**0.0049**
α-Tocotrienol				
Tertile 1 (≤0.9 µg/dL)	39 (25.0)	44 (37.3)	1.00 (Ref)	1.00 (Ref)
Tertile 2 (0.9–1.5 µg/dL)	35 (22.4)	35 (29.7)	1.13 (0.60–2.13)	1.07 (0.49–2.41)
Tertile 3 (>1.5 µg/dL)	82 (52.6)	39 (33.1)	2.37 (1.33–4.22)	2.76 (1.30–5.87)
		*P* for trend ^b^	**0.0024**	**0.0066**

^a^ Multiple logistic regression model adjusted for age, education, family cancer history, BMI, sunburn exposure, skin colour, and eye colour. CI, confidence interval; OR, odds ratio. ^b^ *P*-trend was from the test for linear trends across tertiles of serum micronutrient concentrations based on an ordinal variable according to rank, from lowest to highest. Bold values refer to statistically significant results with *p* < 0.05.

**Table 4:** Effect modification by lifestyle factors on association between vitamin A and E and prostate cancer risk.

Smoking Status	Never-Smokers (*n* = 152)			Ever-Smokers (*n* = 120)			
	Cases, *n* (%)	Controls, *n* (%)	OR (95%) CI ^a^	Cases, *n* (%)	Controls, *n* (%)	OR (95%) CI ^a^	*P* for Interaction ^b^
Lutein							
≤Median of 11.8 µg/dL	18 (20.5)	32 (50.0)	1.00 (Ref)	7 (10.3)	27 (51.9)	1.00 (Ref)	**0.011**
>Median of 11.8 µg/dL	70 (79.5)	32 (50.0)	2.75 (1.12–6.73)	61 (89.7)	25 (48.1)	6.43 (1.62–25.50)	
β-Cryptoxanthin							
Tertile 1 (≤ 7.8 µg/dL)	21 (23.9)	23 (35.9)	1.00 (Ref)	11 (16.2)	18 (34.6)	1.00 (Ref)	**0.0054**
Tertile 2 (7.8–16.0 µg/dL)	28 (31.8)	20 (31.2)	1.18 (0.41–3.35)	25 (36.8)	17(32.7)	6.81 (1.37–33.90)	
Tertile 3 (>16.0 µg/dL)	39 (44.3)	21 (32.8)	1.08 (0.40–2.95)	32 (47.1)	17 (32.7)	7.50 (1.43–39.50)	
β-Carotene							
≤Median of 4.2 µg/dL	22 (25.0)	32 (50.0)	1.00 (Ref)	9 (13.2)	26 (50.0)	1.00 (Ref)	**0.011**
>Median of 4.2 µg/dL	66 (75.0)	32 (50.0)	1.88 (0.80–4.41)	59 (86.8)	26 (50.0)	5.61 (1.54–20.40)	
**Alcohol consumption status**	**Non-regular alcohol drinkers (*n* = 131)**			**Regular alcohol drinkers (*n* = 133)**			
	**Cases, *n* (%)**	**Controls, *n* (%)**	**OR (95%) CI ^a^**	**Cases, *n* (%)**	**Controls, *n* (%)**	**OR (95%) CI ^a^**	***P* for interaction ^b^**
Lutein							
≤Median of 11.8 µg/dL	19 (23.5)	25 (50.0)	1.00 (Ref)	12 (16.9)	31 (50.0)	1.00 (Ref)	**0.036**
>Median of 11.8 µg/dL	62 (76.5)	25 (50.0)	1.81 (0.57–5.76)	59 (83.1)	31 (50.0)	2.71 (1.04–7.10)	
β-Cryptoxanthin							
≤Median of 11.0 µg/dL	39 (48.1)	25 (50.0)	1.00 (Ref)	15 (21.1)	32 (51.6)	1.00 (Ref)	**0.044**
>Median of 11.0 µg/dL	42 (51.9)	25 (50.0)	1.28 (0.44–3.72)	56 (78.9)	30 (48.4)	2.96 (1.15–7.61)	
Ubiquinone							
Tertile 1 ≤ (29.3 µg/dL)	17 (21.0)	17 (34.0)	1.00 (Ref)	16 (22.5)	21 (33.9)	1.00 (Ref)	**0.030**
Tertile 2 (29.3–41.2 µg/dL)	36 (44.4)	16 (32.0)	1.53 (0.42–5.60)	14 (19.7)	20 (32.3)	0.82 (0.24–2.84)	
Tertile 3 (>41.2 µg/dL)	28 (34.6)	17 (34.0)	1.99 (0.48–8.23)	41 (57.7)	21 (33.9)	3.35 (1.10–10.20)	
γ-Tocotrienol							
Tertile 1 (≤0.9 µg/dL)	22 (27.2)	18 (36.0)	1.00 (Ref)	26 (36.6)	23 (37.1)	1.00 (Ref)	**0.026**
Tertile 2 (0.9–1.5 µg/dL)	16 (19.8)	16 (32.0)	2.42 (0.59–9.87)	11 (15.5)	22 (35.5)	0.47 (0.14–1.61)	
Tertile 3 (>1.5 µg/dL)	43 (53.1)	16 (32.0)	2.71 (0.73–9.99)	34 (47.9)	17 (27.4)	3.39 (1.08–10.6)	
α-Tocotrienol							
Tertile 1 (≤0.9 µg/dL)	20 (24.7)	18 (36.0)	1.00 (Ref)	19 (26.8)	25 (40.3)	1.00 (Ref)	**0.021**
Tertile 2 (0.9–1.5 µg/dL)	14 (17.3)	16 (32.0)	0.47 (0.11–2.03)	21 (29.6)	16 (25.8)	1.58 (0.52–4.82)	
Tertile 3 (>1.5 µg/dL)	47 (58.0)	16 (32.0)	1.46 (0.41–5.13)	31 (43.7)	21 (33.9)	3.06 (1.03–9.10)	

^a^ Multiple logistic regression model adjusted for age, education, family cancer history, BMI, sunburn exposure, skin colour, and eye colour. CI, confidence interval; OR, odds ratio. ^b^ *P*-interaction term was from cross-product term of each serum micronutrient concentration (on a continuous scale) and the modifier variable (ever-smoker vs. never smoker, regular alcohol drinker vs. non-regular alcohol drinker) in the regression model.

**Table 5:** Associations between weighted quantile sum indices of micronutrient groups with prostate cancer risk.

	OR (95% CI) ^a^
Vitamin A and provitamin A carotenoids index	1.65 (1.36–1.99)
Vitamin E index	1.47 (1.23–1.75)

^a^ Weighted quantile sum regression model adjusted for age, education, family cancer history, BMI, sunburn exposure, skin colour, and eye colour; ORs are estimated for every decile increase in group index concentrations. CI, confidence interval; OR, odds ratio.

## Data Availability

The data presented in this study are available on request from the corresponding author. The data are not publicly available due to privacy or ethical restrictions.

## Supplementary Materials

The following supporting information can be downloaded at: https://www.mdpi.com/article/10.3390/nu15122677/s1, Table S1: Other clinical characteristics of cases and controls; Table S2: Associations between serum vitamin A and E forms and prostate cancer risk further adjusted for energy intake; Table S3: Associations between serum vitamin A and E forms and prostate cancer risk by tumor grade; Table S4: Associations between serum vitamin A and E forms and prostate cancer by tumor stage; Table S5: Spearman’s correlation coefficients between serum concentrations of micronutrients.

## Abbreviations

BMI, body mass index; OR, odds ratio; PCPT; Prostate Cancer Prevention Trial; SPCS, Singapore Prostate Cancer Study; WQS, weighted quantile sum.
