# Supplementary material for: Association between Vitamin A and E Forms and Prostate Cancer Risk in the Singapore Prostate Cancer Study
Source: Nutrients. 2023 Jun 8;15(12):2677. doi: 10.3390/nu15122677 (PMC10301520; doi:10.3390/nu15122677)
Supplement: Supplementary file 1 [file nutrients-15-02677-s001.zip › nutrients-2398462-supplementary.pdf]

Supplementary Table S1. Other clinical characteristics of cases and controls

		Cases	Controls
Grade[a], n (%)			
	Low	62 (39.7%)	-
	High	94 (60.3%)	-
Stage[b], n (%)			
	Localized	119 (76.3%)	-
	Advanced	37 (23.7%)	-
PSA (ng/mL), Median (IQR)		4.32 (7.72)	1.35 (2.88)

[a] Grade of prostate cancer comprised two categories: ‘Low grade’ (Gleason score ≥ 7) and ‘High grade’ (Gleason score <7).

[b] Stage of prostate cancer was based on the sixth edition of the TNM (tumor–node– metastasis) staging system, with cases being classified as ‘Localized’ (T1 or T2, N0, M0) or ‘Advanced’ (a) M0, T3/T4 & N0; (b) T1-T4 with any N; (c) any T, any N and (d) M1, M1b, M1c).

[c] For 45 cases, the measurement of PSA was not available. For 47 controls, the measurement of PSA was not available.

Supplementary Table S2. Associations between serum vitamin A and E forms and prostate cancer risk further adjusted for energy intake

	Cases (*n*=156)	Controls (*n*=118)		
	n (%)	*n* (%)	Crude OR (95% CI)	Adjusted OR (95% CI)[a]
Retinol				
≤ Median of 60.9 μg/dL	20 (12.8)	59 (50)	1.00 (Ref)	1.00 (Ref)
> Median of 60.9 μg/dL	136 (87.2)	59 (50)	6.80 (3.76–12.30)	6.09 (2.79–13.30)
Lutein				
≤ Median of 11.8 μg/dL	34 (21.8)	59 (50)	1.00 (Ref)	1.00 (Ref)
> Median of 11.8 μg/dL	122 (78.2)	59 (50)	3.59 (2.12–6.06)	2.02 (1.03–3.94)
Zeaxanthin				
Tertile 1 (≤ 3.3 μg/dL)	34 (21.8)	41 (34.7)	1.00 (Ref)	1.00 (Ref)
Tertile 2 (3.3–4.9 μg/dL)	67 (42.9)	38 (32.2)	2.13 (1.16–3.89)	1.39 (0.64–3.02)
Tertile 3 (> 4.9 μg/dL)	55 (35.3)	39 (33.1)	1.70 (0.92–3.14)	1.22 (0.54–2.77)
		P for trend	0.11	0.64
α-Cryptoxanthin				
Tertile 1 (≤ 44.7 μg/dL)	45 (28.8)	40 (34.2)	1.00 (Ref)	1.00 (Ref)
Tertile 2 (44.7–82.9 μg/dL)	43 (27.6)	38 (32.5)	1.01 (0.55–1.85)	1.22 (0.55–2.73)
Tertile 3 (> 82.9 μg/dL)	68 (43.6)	39 (33.3)	1.55 (0.87–2.77)	1.94 (0.91–4.14)
		P for trend	0.13	0.081
β-Cryptoxanthin				
Tertile 1 (≤ 7.8 μg/dL)	29 (18.6)	40 (33.9)	1.00 (Ref)	1.00 (Ref)
Tertile 2 (7.8–16.0 μg/dL)	56 (35.9)	39 (33.1)	1.98 (1.06–3.71)	1.71 (0.75–3.86)
Tertile 3 (>16.0 μg/dL)	71 (45.5)	39 (33.1)	2.51 (1.35–4.66)	1.92 (0.86–4.27)
		P for trend	0.0045	0.12
α-Carotene				
≤ Median of 0.50 μg/dL	64 (41)	86 (72.9)	1.00 (Ref)	1.00 (Ref)
> Median of 0.50 μg/dL	92 (59)	32 (27.1)	3.86 (2.31–6.47)	3.25 (1.66–6.36)
β-Carotene				
≤ Median of 4.2 μg/dL	30 (19.2)	60 (50.8)	1.00 (Ref)	1.00 (Ref)
> Median of 4.2 μg/dL	126 (80.8)	58 (49.2)	4.34 (2.54–7.44)	3.00 (1.51–5.96)
Lycopene				
Tertile 1 (≤3.9 μg/dL)	47 (30.3)	40 (34.5)	1.00 (Ref)	1.00 (Ref)
Tertile 2 (3.9–7.5 μg/dL)	44 (28.4)	38 (32.8)	0.99 (0.54–1.8)	1.01 (0.46–2.24)
Tertile 3 (>7.5 μg/dL)	64 (41.3)	38 (32.8)	1.43 (0.80–2.57)	0.92 (0.43–1.98)
		P for trend	0.22	0.84
Ubiquinone				
Tertile 1 ≤ (29.3 μg/dL)	32 (20.5)	40 (34.5)	1.00 (Ref)	1.00 (Ref)
Tertile 2 (29.3–41.2 μg/dL)	45 (28.8)	37 (31.9)	1.52 (0.80–2.87)	1.21 (0.54–2.72)
Tertile 3 (>41.2 μg/dL)	79 (50.6)	39 (33.6)	2.53 (1.39–4.63)	2.31 (1.06–5.02)
		P for trend	0.0022	**0.030**
δ-Tocopherol				
Tertile 1 (≤ 9.7 μg/dL)	59 (37.8)	40 (33.9)	1.00 (Ref)	1.00 (Ref)

Tertile 2 (9.7–13.1 µg/dL)	45 (28.8)	39 (33.1)	0.78 (0.43–1.41)	0.92 (0.43–1.96)
Tertile 3 (>13.1 µg/dL)	52 (33.3)	39 (33.1)	0.90 (0.51–1.61)	1.13 (0.53–2.4)
		P for trend	0.72	0.78
γ-Tocopherol				
Tertile 1 (≤ 47.2 µg/dL)	54 (34.6)	41 (34.7)	1.00 (Ref)	1.00 (Ref)
Tertile 2 (47.2–74.9 µg/dL)	66 (42.3)	38 (32.2)	1.32 (0.75–2.33)	1.77 (0.82–3.84)
Tertile 3 (>74.9 µg/dL)	36 (23.1)	39 (33.1)	0.70 (0.38–1.29)	0.63 (0.29–1.38)
		P for trend	0.30	0.29
α-Tocopherol				
Tertile 1 (≤1240 µg/dL)	26 (16.7)	40 (33.9)	1.00 (Ref)	1.00 (Ref)
Tertile 2 (1240–1560 µg/dL)	51 (32.7)	39 (33.1)	2.01 (1.05–3.84)	1.6 (0.7–3.67)
Tertile 3 (>1560 µg/dL)	79 (50.6)	39 (33.1)	3.12 (1.67–5.82)	2.37 (1.05–5.34)
		P for trend	0.00041	**0.038**
δ-Tocotrienol				
Tertile 1 (≤ 0.7 µg/dL)	26 (16.7)	42 (35.6)	1.00 (Ref)	1.00 (Ref)
Tertile 2 (0.7–1.1 µg/dL)	39 (25)	43 (36.4)	1.47 (0.76–2.82)	1.33 (0.58–3.06)
Tertile 3 (>1.1 µg/dL)	91 (58.3)	33 (28)	4.45 (2.37–8.37)	3.54 (1.58–7.92)
		P for trend	0.0013	**0.0013**
γ-Tocotrienol				
Tertile 1 (≤ 0.9 µg/dL)	43 (27.6)	41 (34.7)	1.00 (Ref)	1.00 (Ref)
Tertile 2 (0.9–1.5 µg/dL)	38 (24.4)	43 (36.4)	0.84 (0.46–1.55)	0.99 (0.44–2.19)
Tertile 3 (>1.5 µg/dL)	75 (48.1)	34 (28.8)	2.10 (1.17–3.79)	2.95 (1.35–6.48)
		P for trend	0.0098	**0.0064**
α-Tocotrienol				
Tertile 1 (≤ 0.9 µg/dL)	39 (25)	44 (37.3)	1.00 (Ref)	1.00 (Ref)
Tertile 2 (0.9–1.5 µg/dL)	35 (22.4)	35 (29.7)	1.13 (0.6–2.13)	1.05 (0.46–2.37)
Tertile 3 (>1.5 µg/dL)	82 (52.6)	39 (33.1)	2.37 (1.33–4.22)	2.57 (1.20–5.50)
		P for trend	0.0024	**0.013**

[a] Multiple logistic regression model adjusted for age, education, family cancer history, BMI, sunburn exposure, skin colour, eye colour and estimated daily energy intake. CI, confidence interval; OR, odds ratio.

Supplementary Table S3. Associations between serum vitamin A and E forms and prostate cancer risk by tumor grade[a]

	Tumor grade of prostate cancer			Tumor grade of prostate cancer	
	Low (*n*=62)	High (*n*=94)	Controls (*n*=124)	Low	High
	n (%)	*n* (%)	*n* (%)	OR (95% CI)[b]	OR (95% CI)[b]
Retinol					
≤ Median of 60.9 µg/dL	11 (17.7)	9 (9.6)	59 (50)	1.00 (Ref)	1.00 (Ref)
> Median of 60.9 µg/dL	51 (82.3)	85 (90.4)	59 (50)	6.24 (2.16–18.00)	7.81 (2.95–20.70)
Lutein					
≤ Median of 60.9 µg/dL	16 (25.8)	18 (19.1)	59 (50)	1.00 (Ref)	1.00 (Ref)
> Median of 60.9 µg/dL	46 (74.2)	76 (80.9)	59 (50)	1.39 (0.56–3.45)	2.80 (1.25–6.25)
Zeaxanthin					
Tertile 1 (≤ 3.3 µg/dL)	16 (25.8)	18 (19.1)	41 (34.7)	1.00 (Ref)	1.00 (Ref)
Tertile 2 (3.3–4.9 µg/dL)	26 (41.9)	41 (43.6)	38 (32.2)	1.40 (0.48–4.09)	1.69 (0.68–4.20)
Tertile 3 (> 4.9 µg/dL)	20 (32.3)	35 (37.2)	39 (33.1)	0.76 (0.26–2.17)	1.48 (0.57–3.9)
			P for trend	0.58	0.45
α-Cryptoxanthin					
Tertile 1 (≤ 44.7 µg/dL)	22 (35.5)	23 (24.5)	40 (34.2)	1.00 (Ref)	1.00 (Ref)
Tertial 2 (44.7–82.9 µg/dL)	14 (22.6)	29 (30.9)	38 (32.5)	0.73 (0.25–2.15)	1.42 (0.55–3.70)
Tertile 3 (> 82.9 µg/dL)	26 (41.9)	42 (44.7)	39 (33.3)	1.34 (0.48–3.72)	2.26 (0.94–5.42)
			P for trend	0.53	0.064
β-Cryptoxanthin					
Tertile 1 (≤ 7.8 µg/dL)	12 (19.4)	17 (18.1)	40 (33.9)	1.00 (Ref)	1.00 (Ref)
Tertile 2 (7.8–16.0 µg/dL)	21 (33.9)	35 (37.2)	39 (33.1)	1.28 (0.42–3.92)	1.81 (0.7–4.70)
Tertile 3 (>16.0 µg/dL)	29 (46.8)	42 (44.7)	39 (33.1)	1.66 (0.57–4.86)	1.94 (0.77–4.85)
			P for trend	0.35	0.18
α-Carotene					
≤ Median of 0.50 µg/dL	24 (38.7)	40 (42.6)	86 (72.9)	1.00 (Ref)	1.00 (Ref)
> Median of 0.50 µg/dL	38 (61.3)	54 (57.4)	32 (27.1)	3.31 (1.35–8.08)	3.78 (1.74–8.21)
β-Carotene					
≤ Median of 4.2 µg/dL	12 (19.4)	18 (19.1)	60 (50.8)	1.00 (Ref)	1.00 (Ref)
> Median of 4.2 µg/dL	50 (80.6)	76 (80.9)	58 (49.2)	3.38 (1.27–9.04)	3.28 (1.44–7.49)
Lycopene					
Tertile 1 (≤3.9 µg/dL)	20 (32.3)	27 (29)	40 (34.5)	1.00 (Ref)	1.00 (Ref)
Tertile 2 (3.9–7.5 µg/dL)	18 (29)	26 (28)	38 (32.8)	0.89 (0.31–2.6)	1.00 (0.39–2.52)
Tertile 3 (>7.5 µg/dL)	24 (38.7)	40 (43)	38 (32.8)	0.75 (0.27–2.07)	1.14 (0.48–2.73)
			P for trend	0.58	0.76
Ubiquinone					
Tertile 1 ≤ (29.3 µg/dL)	15 (24.2)	17 (18.1)	40 (34.5)	1.00 (Ref)	1.00 (Ref)
Tertile 2 (29.3–41.2 µg/dL)	17 (27.4)	28 (29.8)	37 (31.9)	1.15 (0.38–3.49)	1.59 (0.61–4.1)
Tertile 3 (>41.2 µg/dL)	30 (48.4)	49 (52.1)	39 (33.6)	1.55 (0.57–4.21)	2.95 (1.18–7.34)

			P for trend	0.38	**0.018**
δ-Tocopherol					
Tertile 1 (≤ 9.7 μg/dL)	27 (43.5)	32 (34)	40 (33.9)	1.00 (Ref)	1.00 (Ref)
Tertile 2 (9.7–13.1 μg/dL)	17 (27.4)	28 (29.8)	39 (33.1)	1.26 (0.46–3.40)	0.94 (0.39–2.26)
Tertile 3 (>13.1 μg/dL)	18 (29)	34 (36.2)	39 (33.1)	0.85 (0.31–2.29)	1.09 (0.45–2.61)
			P for trend	0.80	0.86
γ-Tocopherol					
Tertile 1 (≤ 47.2 μg/dL)	25 (40.3)	29 (30.9)	41 (34.7)	1.00 (Ref)	1.00 (Ref)
Tertile 2 (47.2–74.9 μg/dL)	24 (38.7)	42 (44.7)	38 (32.2)	1.17 (0.42–3.25)	2.08 (0.83–5.17)
Tertile 3 (>74.9 μg/dL)	13 (21)	23 (24.5)	39 (33.1)	0.36 (0.12–1.09)	0.73 (0.29–1.81)
			P for trend	0.085	0.47
α-Tocopherol					
≤ Median of 1402 μg/dL	25 (40.3)	24 (25.5)	59 (50.0)	1.00 (Ref)	1.00 (Ref)
> Median of 1402 μg/dL	37 (59.7)	70 (74.5)	59 (50.0)	2.00 (0.82–4.87)	2.51 (1.15–5.45)
δ-Tocotrienol					
Tertile 1 (≤ 0.7 μg/dL)	11 (17.7)	15 (16)	42 (35.6)	1.00 (Ref)	1.00 (Ref)
Tertile 2 (0.7–1.1 μg/dL)	13 (21)	26 (27.7)	43 (36.4)	1.30 (0.42–4.06)	1.63 (0.61–4.33)
Tertile 3 (>1.1 μg/dL)	38 (61.3)	53 (56.4)	33 (28)	3.06 (1.05–8.95)	4.25 (1.66–10.90)
			P for trend	**0.031**	**0.0020**
γ-Tocotrienol					
Tertile 1 (≤ 0.9 μg/dL)	15 (24.2)	28 (29.8)	41 (34.7)	1.00 (Ref)	1.00 (Ref)
Tertile 2 (0.9–1.5 μg/dL)	16 (25.8)	22 (23.4)	43 (36.4)	1.31 (0.44–3.92)	0.73 (0.29–1.84)
Tertile 3 (>1.5 μg/dL)	31 (50)	44 (46.8)	34 (28.8)	4.35 (1.48–12.80)	2.14 (0.9–5.10)
			P for trend	**0.0068**	0.088
α-Tocotrienol					
≤ Median of 1.1μg/dL	22 (35.5)	28 (29.8)	63 (53.4)	1.00 (Ref)	1.00 (Ref)
> Median of 1.1μg/dL	40 (64.5)	66 (70.2)	55 (46.6)	2.76 (1.16–6.55)	2.53 (1.22–5.26)

[a] Grade of prostate cancer comprised two categories: ‘Low grade (Gleason score ≥ 7) and ‘High grade’ (Gleason score <7).

[b] Multiple logistic regression model adjusted for age, education, family cancer history, BMI, sun exposure, skin colour, and eye colour. CI, confidence interval; OR, odds ratio.

Supplementary Table S4. Associations between serum vitamin A and E forms and prostate cancer by tumor stage[a]

	Tumor stage of prostate cancer			Tumor stage of prostate cancer	
	Localized (*n*=119)	Advanced (*n*=37)	Controls (*n*=124)	Localized	Advanced
	n (%)	*n* (%)	*n* (%)	OR (95% CI)[a]	OR (95% CI)[a]
Retinol					
≤ Median of 60.9 µg/dL	16 (13.4)	4 (10.8)	59 (50)	1.00 (Ref)	1.00 (Ref)
> Median of 60.9 µg/dL	103 (86.6)	33 (89.2)	59 (50)	6.11 (2.65-14.10)	7.93 (2.11-29.80)
Lutein					
≤ Median of 11.8 µg/dL	25 (21)	9 (24.3)	59 (50)	1.00 (Ref)	1.00 (Ref)
> Median of 11.8 µg/dL	94 (79)	28 (75.7)	59 (50)	2.12 (1.02-4.38)	3.51 (1.11-11.1)
Zeaxanthin					
Tertile 1 (≤ 3.3 µg/dL)	28 (23.5)	6 (16.2)	41 (34.7)	1.00 (Ref)	1.00 (Ref)
Tertile 2 (3.3–4.9 µg/dL)	55 (46.2)	12 (32.4)	38 (32.2)	1.42 (0.621-3.25)	1.56 (0.39-6.26)
Tertile 3 (> 4.9 µg/dL)	36 (30.3)	19 (51.4)	39 (33.1)	1.00 (0.417-2.39)	2.81 (0.71-11.1)
			P for trend	0.97	0.13
α-Cryptoxanthin					
Tertile 1 (≤ 44.7 µg/dL)	33 (27.7)	12 (32.4)	40 (34.2)	1.00 (Ref)	1.00 (Ref)
Tertial 2 (44.7–82.9 µg/dL)	34 (28.6)	9 (24.3)	38 (32.5)	1.38 (0.58-3.27)	0.45 (0.11-1.86)
Tertile 3 (> 82.9 µg/dL)	52 (43.7)	16 (43.2)	39 (33.3)	1.88 (0.82-4.31)	1.81 (0.57-5.7)
			P for trend	0.13	0.28
β-Cryptoxanthin					
Tertile 1 (≤ 7.8 µg/dL)	22 (18.5)	7 (18.9)	40 (33.9)	1.00 (Ref)	1.00 (Ref)
Tertile 2 (7.8–16.0 µg/dL)	39 (32.8)	17 (45.9)	39 (33.1)	1.56 (0.64-3.79)	1.98 (0.56-6.98)
Tertile 3 (>16.0 µg/dL)	58 (48.7)	13 (35.1)	39 (33.1)	2.07 (0.86-4.94)	1.47 (0.41-5.3)

			P for trend	0.11	0.62
α-Carotene					
≤ Median of 0.50 µg/dL	41 (34.5)	23 (62.2)	86 (72.9)	1.00 (Ref)	1.00 (Ref)
> Median of 0.50 µg/dL	78 (65.5)	14 (37.8)	32 (27.1)	4.2 (2.04-8.64)	2.15 (0.73-6.35)
β-Carotene					
≤ Median of 4.2 µg/dL	21 (17.6)	9 (24.3)	60 (50.8)	1.00 (Ref)	1.00 (Ref)
> Median of 4.2 µg/dL	98 (82.4)	28 (75.7)	58 (49.2)	3.33 (1.57-7.05)	3.18 (1.00-10.1)
Lycopene					
Tertile 1 (≤3.9 µg/dL)	32 (27.1)	15 (40.5)	40 (34.5)	1.00 (Ref)	1.00 (Ref)
Tertile 2 (3.9–7.5 µg/dL)	35 (29.7)	9 (24.3)	38 (32.8)	1.19 (0.50-2.83)	0.62 (0.17-2.20)
Tertile 3 (>7.5 µg/dL)	51 (43.2)	13 (35.1)	38 (32.8)	1.04 (0.45-2.38)	0.87 (0.27-2.83)
			P for trend	0.93	0.79
Ubiquinone					
Tertile 1 ≤ (29.3 µg/dL)	25 (21)	7 (18.9)	40 (34.5)	1.00 (Ref)	1.00 (Ref)
Tertile 2 (29.3–41.2 µg/dL)	34 (28.6)	11 (29.7)	37 (31.9)	1.14 (0.47-2.74)	3.70 (0.84-16.30)
Tertile 3 (>41.2 µg/dL)	60 (50.4)	19 (51.4)	39 (33.6)	2.07 (0.91-4.73)	6.57 (1.52-28.50)
			P for trend	0.073	**0.012**
δ-Tocopherol					
Tertile 1 (≤ 9.7 µg/dL)	50 (42)	9 (24.3)	40 (33.9)	1.00 (Ref)	1.00 (Ref)
Tertile 2 (9.7–13.1 µg/dL)	34 (28.6)	11 (29.7)	39 (33.1)	0.93 (0.42-2.08)	1.85 (0.52-6.55)
Tertile 3 (>13.1 µg/dL)	35 (29.4)	17 (45.9)	39 (33.1)	0.97 (0.43-2.19)	2.37 (0.68-8.31)
			P for trend	0.93	0.18

γ-Tocopherol					
Tertile 1 (≤ 47.2 µg/dL)	40 (33.6)	14 (37.8)	41 (34.7)	1.00 (Ref)	1.00 (Ref)
Tertile 2 (47.2–74.9 µg/dL)	53 (44.5)	13 (35.1)	38 (32.2)	2.13 (0.933-4.85)	1.53 (0.44-5.30)
Tertile 3 (>74.9 µg/dL)	26 (21.8)	10 (27)	39 (33.1)	0.627 (0.26-1.49)	0.70 (0.20-2.33)
			P for trend	0.38	0.57
α-Tocopherol					
≤ Median of 1402 µg/dL	40 (33.6)	9 (24.3)	59 (50)	1.00 (Ref)	1.00 (Ref)
> Median of 1402 µg/dL	79 (66.4)	28 (75.7)	59 (50)	1.99 (0.99-4.01)	8.10 (2.2-29.9)
δ-Tocotrienol					
≤ Median of 0.9µg/dL	38 (31.9)	11 (29.7)	64 (54.2)	1.00 (Ref)	1.00 (Ref)
> Median of 0.9µg/dL	81 (68.1)	26 (70.3)	54 (45.8)	1.93 (0.96-3.88)	2.02 (0.74-5.54)
γ-Tocotrienol					
Tertile 1 (≤ 0.9 µg/dL)	34 (28.6)	9 (24.3)	41 (34.7)	1.00 (Ref)	1.00 (Ref)
Tertile 2 (0.9–1.5 µg/dL)	28 (23.5)	10 (27)	43 (36.4)	0.97 (0.40-2.32)	1.09 (0.50-7.15)
Tertile 3 (>1.5 µg/dL)	57 (47.9)	18 (48.6)	34 (28.8)	3.52 (1.49-8.32)	2.99 (0.88-10.10)
			P for trend	**0.0035**	0.078
α-Tocotrienol					
Tertile 1 (≤ 0.9 µg/dL)	31 (26.1)	8 (21.6)	44 (37.3)	1.00 (Ref)	1.00 (Ref)
Tertile 2 (0.9–1.5 µg/dL)	29 (24.4)	6 (16.2)	35 (29.7)	1.16 (0.49-2.73)	0.76 (0.17-3.44)
Tertile 3 (>1.5 µg/dL)	59 (49.6)	23 (62.2)	39 (33.1)	2.53 (1.12-5.72)	4.79 (1.39-16.50)
			P for trend	**0.024**	**0.0088**

[a] Stage of prostate cancer was based on the sixth edition of the TNM (tumor–node– metastasis) staging system, with cases being classified as ‘Localized’ (T1 or T2, N0, M0) or ‘Advanced’ (a) M0, T3/T4 & N0; (b) T1-T4 with any N; (c) any T, any N and (d) M1, M1b, M1c).

[b] Multiple logistic regression model adjusted for age, education, family cancer history, BMI, sun exposure, skin colour, and eye colour. CI, confidence interval; OR, odds ratio.

Supplementary Table S5. Spearman's correlation coefficients between serum concentrations of micronutrients

	Retinol	Lutein	Zeaxanthin	α-Cryptoxanthin	β-Cryptoxanthin	α-Carotene	β-Carotene	Lycopene	Ubiquinone	δ-Tocopherol	γ-Tocopherol	α-Tocopherol	δ-Tocotrienol	γ-Tocotrienol	α-Tocotrienol
Retinol	1.000														
Lutein	**0.35**	1.000													
Zeaxanthin	**0.30**	**0.24**	1.000												
α-Cryptoxanthin	0.12	**0.24**	0.15	1.000											
β-Cryptoxanthin	**0.22**	**0.32**	**0.24**	**0.74**	1.000										
α-Carotene	**0.28**	**0.48**	0.01	**0.25**	**0.35**	1.00									
β-Carotene	**0.33**	**0.56**	0.13	**0.41**	**0.61**	**0.72**	1.000								
Lycopene	**0.18**	0.14	**0.21**	**0.34**	**0.55**	**0.34**	**0.50**	1.00							
Ubiquinone	**0.33**	**0.30**	**0.44**	-0.01	0.13	**0.21**	**0.26**	**0.27**	1.000						
δ-Tocopherol	**0.20**	0.03	**0.23**	-0.06	-0.10	-0.09	-0.09	0.01	**0.40**	1.000					
γ-Tocopherol	0.13	0.11	0.10	0.06	-0.00	0.02	-0.01	0.01	**0.17**	**0.65**	1.000				
α-Tocopherol	**0.46**	**0.30**	**0.33**	**0.16**	**0.25**	**0.20**	**0.31**	**0.19**	**0.54**	**0.35**	**0.23**	1.000			
δ-Tocotrienol	0.24	**0.32**	**0.38**	0.02	0.11	0.14	**0.20**	0.12	**0.37**	**0.20**	0.08	0.16	1.000		
γ-Tocotrienol	0.19	**0.23**	**0.25**	-0.02	-0.00	0.04	0.07	-0.06	**0.34**	**0.49**	**0.18**	**0.24**	**0.51**	1.000	
α-Tocotrienol	0.17	**0.18**	**0.30**	-0.05	-0.06	0.07	0.07	0.05	**0.35**	**0.48**	0.11	**0.18**	**0.46**	**0.67**	1.000

Correlations that are statistically significant (*P*-value < 0.01) are in bold.
